# Properties and Structure of Concretes Doped with Production Waste of Thermoplastic Elastomers from the Production of Car Floor Mats

**DOI:** 10.3390/ma14040872

**Published:** 2021-02-11

**Authors:** Malgorzata Ulewicz, Alina Pietrzak

**Affiliations:** Faculty of Civil Engineering, Czestochowa University of Technology, Dabrowskiego 69 Street, PL 42-201 Czestochowa, Poland; alina.pietrzak@pcz.pl

**Keywords:** waste thermoplastic elastomer, concrete, compression strength, frost resistance, microstructure

## Abstract

This article presents physical and mechanical properties of concrete composites that include waste thermoplastic elastomer (TPE) from the production process of car floor mats. Waste elastomer (2–8 mm fraction) was used as a substitute for fine aggregate in quantities of 2.5, 5.0, 7.5, and 10% of the cement weight. For all series of concrete, the following tests were carried out: compression strength, bending tensile strength, splitting tensile strength, absorbability, density, resistance to water penetration under pressure, frost resistance, and abrasion resistance, according to applicable standards. Moreover, SEM/EDS analysis was carried out on the surface microstructure of synthesized concrete composites. It was proven that the use of production waste from the production process of car floor mats in the quantity of 2.5% does not influence the change of the concrete microstructure and it does not result in the decrease of the mechanical parameters of concrete modified with waste. All concrete modified with the addition of waste meet standards requirements after carrying out 15 cycles of freezing and thawing, and the average decrease in compression strength did not exceed 20%. Adding waste in the quantity of 2.5% allows for limiting the use of aggregate by about 5%, which is beneficial for the natural environment.

## 1. Introduction

Concrete, which is a composite material with a cement matrix, is commonly used in the construction industry. A systematic increase in the quantity of manufactured concrete causes the use of large quantities or natural aggregates, and the excessive use of natural resources results in environment degradation. Therefore, in recent years, many research centers have carried out tests concerning the possibility of using various waste materials, such as domestic and industrial waste [1], bottom ash [2], sanitary and utilitarian ceramic [3,4], cathode ray tube glass (CRT ) [5], recycled glass sand aggregates [6], and slag aggregate [7]. Amongst various waste materials, there is a large focus on polymer waste [8,9,10,11]. According to the literature, one of the most tested materials in terms of waste use for the said purpose is polyethylene terephthalate (PET) used as aggregate [12,13,14,15,16] or fiber [17,18,19]. Recyclate obtained from PET waste has been used in concrete as the substitute for aggregate or in the form of fiber as reinforcement. The manner of PET recyclate preparation and its form have a large impact on the parameters of manufactured concrete. Recyclate with a smooth, spherical surface has less impact on the concrete workability than recyclate with an uneven form. As proven by most authors, adding waste PET to concrete results in decreasing compression strength, tensile strength, and bending strength of concrete, as well as the modulus of elasticity, irrespective of tested consistency and water–cement ratio.

Moreover, tests concerning the use of waste recyclate from polyethylene and polypropylene [20,21,22,23] as the substitute for aggregate or as the reinforcement of concrete have also been carried out. Recycling mixtures of this material are characterized with various quality and mechanical properties [24,25]. Due to the fact that the properties of fibers from pure synthetic material are significantly different than the properties of fibers obtained from recycling material, fibers made of pure polypropylene or polyethylene are more often used in tests [26,27,28]. Concrete with polypropylene fibers in the quantity up to 1% present higher compression strength and splitting or bending strength than concrete that does not contain such material (standard concrete).

An increase in the quantity of synthetic fibers above such level results in the worsening of the mechanical properties of modified concrete. There is also much information on the use of waste polystyrene [29,30,31,32] and rubber [33,34,35,36,37,38,39,40,41,42,43] to manufacture concrete. Along with the increase in the quantity of rubber waste used as a substitute for sand, concrete mixtures are characterized with lower [36,37,38,39,40,41] or higher [34] workability. Concrete containing rubber recyclate presents lower values of mechanical parameters than concrete manufactured without the addition of waste [35,36]. On the other hand, the use of rubber recyclate together with waste glass powder or silica sand has a positive impact on the mechanical properties of manufactured concrete [44,45]. The mechanical properties of concretes can be improved, according to literature reports, by introducing polymer, sisal, or steel fibers into the concrete [46,47,48]. The compressive, tensile, and bending strength of concrete composites increases in comparison to normal concretes with an increase in the quantity of fibers amount.

Currently, a large quantity of polymer waste materials is available on the market, which are of no practical use. However, it is worth testing them to possibly use them in the production process of composite materials. This group covers waste thermoplastic elastomer from the production process of car floor mats. According to the waste catalogue [49], waste from the production of car floor mats is classified as waste group 12 01 05. Car floor mats are usually made of needle velour (polypropylene or polyamide) finished with rubber crumbs (e.g., polybutadiene or polyolefin). Moreover, car floor mats are also manufactured on the basis of polyethylene terephthalate and polyethylene–polypropylene copolymer. What is more, this material contains modifying additives, such as flame retardant agent, mineral fillings, reinforcing fillings, plasticizer, UV stabilizers, antioxidants, and coloring agents. The diverse composition of modifying additives and the diverse chemical structure of the main polymer makes segregation of this type of waste ineffective, and renders recycling impossible. Thus, the search for new possibilities for the use of waste thermoplastic elastomer from the production process of car floor mats is an essential topic due to environmental protection.

## 2. Materials and Methods

### 2.1. Materials

**Figure 1:**
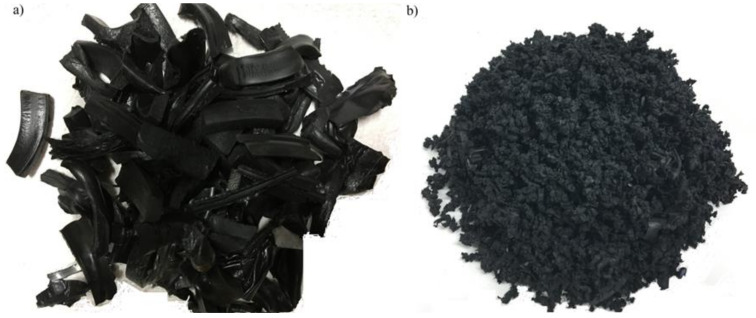
Production waste from the production process of car floor mats: (**a**) initially crushed material; (**b**) material crushed to 2–8 mm fraction.

The following concrete was used for the tests: Portland cement CEM I 42.5R (Cemex, Rudniki, Poland), which meets the requirements of standard PN-EN 197-1, sand, gravel aggregate with 2–8 mm and 8–16 mm fractions, water, additives of CHRYSTO Plast 331, and CHRYSTO Air LB (the company CHRYSTO, Błonie, Poland), as well as waste thermoplastic elastomers from the production process of car floor mats at the production company Gumotest (Bielsko Białą, Poland). The waste used in this research was a thermoplastic elastomer (TPE-V; Bielsko Biała, Poland) based on an ethylene propylene diene blend (EPDM). Following initial crushing, production waste (Figure 1) was crushed into a 2–8 mm fraction in a granulator (SG-2417 SHINI, Donggung, China). With the use of the X-ray spectrometer WDXRF (Model S8 Tiger of the company Bruker, Billerica, USA), the elemental composition of the waste thermoplastic elastomer used in the tests was identified, which is presented in Table 1. For the tests, tap water from the water intake in Czestochowa was used, which meets the requirements of standard PN-EN 1008:2004 [50]. The average quantity of nitrate in the water was 37.0 mg/dm^3^, the average quantity of chlorides in the. water was 31.5 mg/dm^3^, and the average quantity of sulphates in the water was 53.8 mg/dm^3^.

**Figure 2:**
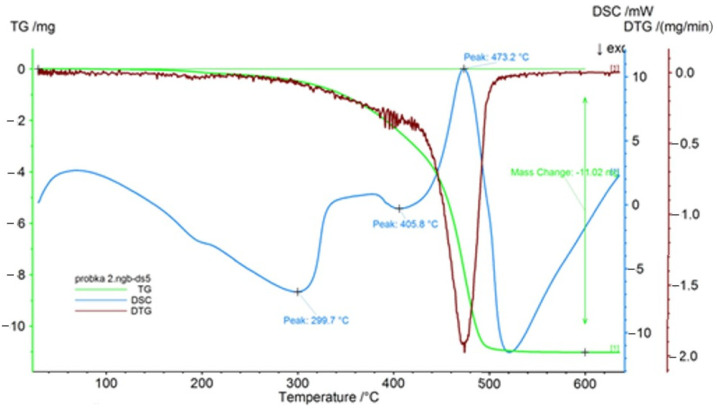
TGA−DTA thermogram for the waste thermoplastic elastomer.

TGA-DTA analysis was carried out for the waste used in the tests. The test was carried out in the thermal analysis device Jupiter STA 449 F5 (of the company Netzsch, Netzsch, Selb, Germany) in the temperature range from 30 to 600 °C, with the temperature increase rate 10 °C/min in an air atmosphere, and with the gas flow rate 100 cm^3^/min. The results of thermogravimetric analysis (TG), differential thermogravimetric analysis (DTG), and differential scanning calorimetry (DSC) are presented in Figure 2. A loss of weight of the samples can be seen in a wide range of temperature from ca. 200 to 500°C, and is ca. 11%. The processes connected with weight loss are endothermic.

### 2.2. Methods

Five series of concrete were prepared. To design the composition of the concrete mixture of the reference concrete (RC), an experimental method developed by Professor Kuczyński was used. It is the most popular Polish computational–experimental method of designing a concrete mix called the method of successive approximations or starting with iteration. It involves the calculation of three unknowns, i.e., the amount of cement, water, and aggregate, by using three basic equations: strength, tightness, and water demand (consistency). The obtained results are verified experimentally. The water–cement ratio was assumed to be 2:22 and the consistency class was assumed to be S2. To prepare the reference concrete, Portland cement CEM I 42.5R, the mixture of gravel–sand aggregates with a particle size distribution of 0–16 mm and a sand content of 33.1%, tap water and plasticizing admixture CHRYSTO Plast 331 in the quantity of 0.35% of the cement weight, and air entraining admixture CHRYSTO Air LB in the quantity of 0.2% of the cement weight were used. The reference concrete (RC) was modified with waste thermoplastic elastomer from the production process of car floor mats. The waste was added to concrete in the quantities of 2.5%, 5.0%, 7.5%, and 10% of the cement weight, adjusting the volume of gravel aggregate with a particle size distribution of 2–8 mm. The series of concrete were identified as S1-1, S1-2, S1-3, and S1-4, accordingly, and the composition of the designed mixtures is presented in Table 2. The waste thermoplastic elastomers from the production process of car floor mats were added directly to the mixer and pre-mixed with the aggregate. The workability of the mixture modified with waste did not change. The samples were shaped in accordance with the applicable standards, and then disassembled after 24 h and stored at a temperature of 18 ± 2 °C and relative humidity above 90%.

For all concrete mixtures, the consistency was defined according to standard PN-EN 12350-2:2011 [51], and the air quantity was defined according to standard PN-EN 12350-7:2011 [52]. On the other hand, concrete was tested for compression strength after 7, 28, and 56 days of the curing period according to standards PN-EN 206-1+A1:2016-12, PN-EN 12390-1:2013-03, PN-EN 12390-2:2019-07, PN-EN 12390-3:2019-07, and PN-EN 12390-4:2001 [53,54,55,56,57], and the following tests were carried out: bending tensile strength according to standard PN-EN 12390-5:2019-08 [58], and splitting tensile strength according to standard PN-EN 12390-6:2011 [59]. What is more, concrete absorbability was tested according to standard PN-B 06250 [60], density was tested according to standard PN-EN 206-1+A1:2016-12 [53], and resistance to water penetration under pressure was tested according to PN-EN 12390-8:2019-08 [61]. In addition, frost resistance was identified for the tested concretes: decrease in resistance and weight loss after frost resistance test according to standard PN-B 06250 [61], and abrasion resistance according to standard PN-EN 13892-3:2015 [62].

SEM/EDS analysis of the microstructure of the synthesized concrete composites that included the waste thermoplastic elastomer was also carried out. Scanning electron microscopy (SEM), LEO Electron Microscopy Ltd., Cambridge, UK) was used for the tests, which was equipped with a system for chemical composition analysis based on X-ray energy dispersion—EDS (energy dispersive spectroscopy, Bruker AXS, Karlsruhe, Germany).

## 3. Results and Discussion

For the concrete mixture of the reference concrete, an S2 consistency was assumed (concrete slump at 50–90 mm). After preparation of the concrete mixture, samples were collected to define the consistency class and air content in the concrete mixture. For the reference mixture (RC), the slump was 70 mm, which corresponds to the S2 class. Air content in this mixture was 3.5%. For concrete mixtures modified with the waste thermoplastic elastomer (S1-1–S1-4), the slump was between 50 and 75 mm, which also corresponds to the S2 consistency class (Table 3). The air content in the concrete mixtures modified with production waste was at the level of 3.65–4.1%.

For each series of concrete, 18 cubic samples with a 150 mm side were prepared, which were subject to compression strength tests after 7, 28, and 56 days of the curing period under laboratory conditions. For the results obtained in the individual series, the standard deviation and confidence level at 95% were identified. The reference concrete (RC) after seven days of curing was characterized with an average compression strength of 46.6 ± 0.95 MPa, whilst after 28 days it was 57.0 ± 1.59 MPa, and 61.9 ± 1.19 MPa after 56 days (Table 4). The average compression strengths for the concretes modified with the waste thermoplastic elastomer in the quantity of 2.5% after seven days of curing period were at the level of the average compression strength of the reference concrete. On the other hand, the average compression strength of the concretes containing the waste elastomer in the quantities of 5.0%, 7.5%, and 10% was lower than in the case of the reference concrete. Therefore, from the point of view of compression strength tested after seven days of the curing period, only the use of the waste thermoplastic elastomer in the quantity below 2.5% does not cause a significant decrease in this parameter. The average compression strength for the concrete series S1-1 containing 2.5% of waste tested after 28 days of the curing period was at the level of the reference concrete (57.8 ± 0.36 MPa), whilst the other series of concrete (S1-2, S1-3, and S1-4) modified with waste presented a lower 28-day average compression strength than the reference series concrete. The decrease in the average compression strength for the individual concrete series was between 9.6% and 22.6%. After 56 days of the sample curing period, the reference concrete (RC) obtained an average compression strength higher by 8.5% than the 28-day average compression strength, which was 61.9 ± 1.19 MPa. In the event of the concrete series modified with waste, the compression strength increased compared to the strength obtained after 28 days. Concrete series S1-1 containing 2.5% waste obtained strength (62.1 ± 1.7 MPa) at the level of the reference concrete, whilst the other series of concrete, which are S1-2, S1-3, and S1-4, obtained lower average compression strengths compared to the reference concrete—from 11.3% to 24.3%.

The addition of the waste thermoplastic elastomer to concrete also influenced the bending strength and splitting tensile strength of the concrete (Table 5). Adding production waste, which was used as a substitute for aggregate with particle size distribution 2–8 mm, at the quantities of 2.5% and 5.0% to concrete had a positive impact on the tested parameters. For series S1-1 and S1-2, an increase in the average bending strength by 3.1% and 2.2%, accordingly, as compared to the reference concrete, was noted. For the other series of modified concrete (S1-3 and S1-4), a slight decrease in the average bending strength by 0.8% and 1.2%, accordingly, as compared to the reference concrete series, was noted. On the other hand, the average splitting tensile strength of the reference concrete was equal to 3.78 ± 0.98 MPa. In the event of concrete containing the addition of production waste in the quantities of 2.5% and 5.0% (series S1-1 and S1-2), an increase of the tested parameter by 13.5% and 9.0%, accordingly, as compared to the RC series, was noted. Meanwhile, in the event of other series (S1-3 and S1-4), a decrease in the average splitting tensile strength of concrete by 5.3% and 12.2%, accordingly, as compared to the reference series, was noted. The increase in bending tensile strength and splitting tensile strength is most likely related to the grain surface of the waste thermoplastic elastomer granulate and the surface of the natural aggregate grain. Both sand and gravel are natural pebble aggregates with a very smooth surface, while the waste granulate used had a much rougher surface, which perhaps resulted in better adhesion with the cement matrix and increased the mechanical properties of the concrete.

The next stage of tests was to determine the absorbability, density, water penetration under pressure, frost resistance, and abrasion resistance of the concrete (Table 6). The absorbability test was carried out after 28 days of curing the samples. In the event of the reference concrete (RC), the absorbability was 5.4%. A similar level of absorbability (from 4.9% to 5.6%) was presented by the concrete modified with production waste in the quantities of 2.5%, 5.0%, 7.5%, and 10%. According to standard PN-B 06250 [56], the absorbability of concrete exposed to environmental factors should not be higher than 5%, whilst concrete covered from the direct influence of environmental factors should not exceed 9%. Both the reference series concrete, as well as the concrete modified with the waste thermoplastic elastomer, obtained absorbability below 9%. This means that concrete manufactured with this type of production waste should be covered from the direct influence of environmental factors. Due to its density, which was 2271 kg/m^3^, the reference concrete was classified into a standard concrete category according to standard PN-EN 12390-4:2001 [53]. Similarly, all concretes modified with the waste thermoplastic elastomer were included in the standard concrete category, since their density was between 2000 and 2600 kg/m^3^. The reference concrete obtained an average depth of water penetration equal to 65 mm. For the series of concrete modified with the waste thermoplastic elastomer, the average depth of water penetration was from 60 to 67 mm. The lowest value of the tested parameter (60 mm) was obtained for series S1-1, in which 2.5% of production waste was used, whilst the highest value (67 mm) was obtained for concrete series S1-4, in which 10% of the said waste was used. In the event of the reference concrete (RC), the abrasion strength was 7.4 cm^3^/50 cm^2^. For the concrete modified with the waste of thermoplastic elastomer, a lower abrasion strength was obtained than for the reference series concrete—it was 2.7–6.8%.

**Figure 3:**
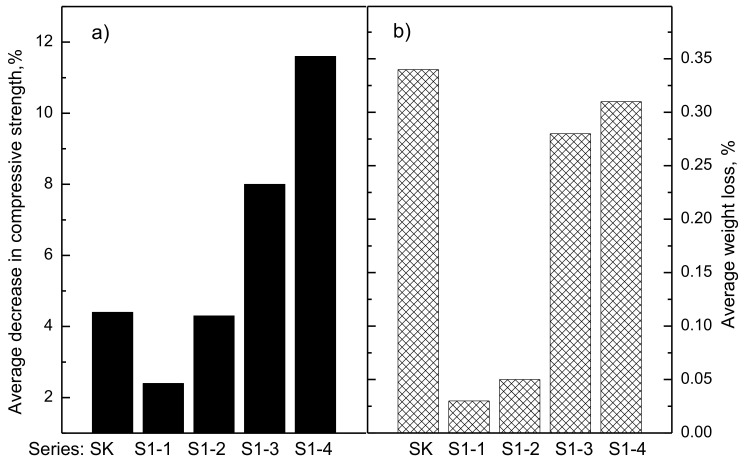
The average decrease in compression strength of the concrete after the frost strength test (**a**) and the average weight drop (**b**), both in %.

A frost resistance test was carried out for all series of concrete. For each series of concrete, 12 cubic samples with a 100 mm side were prepared, of which six samples were subject to frost resistance tests, and six samples were left in water as the reference samples. In the event of the reference concrete, the average decrease in compression strength after 150 cycles of freezing and thawing was 4.4%, and the average weight loss was 0.34% (Figure 3).

In the event of the concrete modified with the waste thermoplastic elastomer used as the substitute for gravel aggregate with particle size distribution 2–8 mm in the quantities of 2.5% and 5.0% of the cement weight, a lower average decrease in the compression strength than for the reference series concrete was noted. The average compression strength of the concrete after the frost resistance tests decreased, along with an increase of the amount of production waste added to the concrete. The lowest decrease in the average compression strength after the frost resistance test was noted for concrete series with the addition of 2.5% production waste, where it was 2.4%, whilst the highest (11.6%) was noted for concrete containing 10% of production waste. After the frost resistance test, the average weight loss was at the level of 0.05–0.34%. None of the samples cracked and there were no scratches.

**Figure 4:**
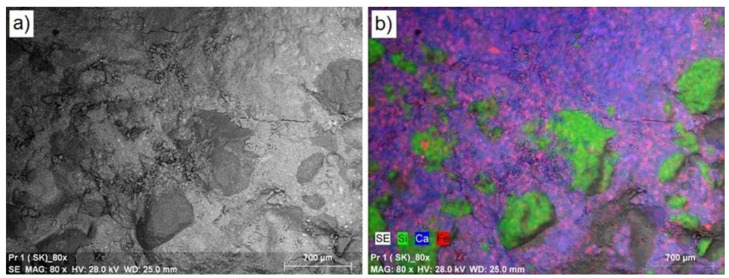
Microstructure of the controlled series concrete: (**a**) enlarged by 80×; (**b**) location map of the dominant elements in a given area.

**Figure 5:**
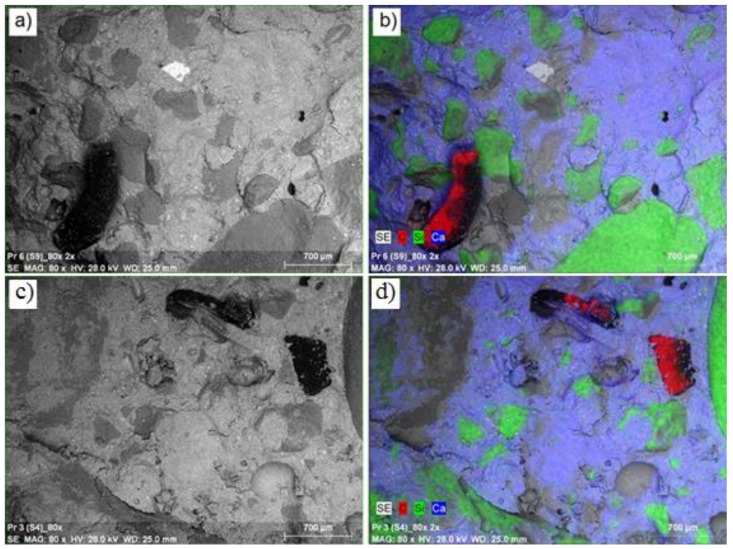
Concrete microstructure: (**a**) reference series S1-1 enlarged by 80×; (**b**) location map of the dominant elements in a given area, concrete series S1-1; (**c**) series S1-4 enlarged by 80×; (**d**) location map of the dominant elements in a given area, concrete series S1-4.

During the next stage of tests, analysis of the surface layer of the synthesized composites was carried out, mainly defining their morphology and elemental composition. Figure 4 presents a microscopic image for the reference concrete (RC) enlarged 80 times (Figure 4a), together with location maps of the dominant elements in this area (Figure 4b). In the reference concrete (RC), a lighter structure of concrete matrix is visible, as well as a darker area, which represents the aggregate based on silicon. The microstructure at the border of the aggregate with the cement matrix is of high density. According to EDS analysis of concrete surface visible on the image, in addition to calcium (33.06%; blue color), significant amounts of silicon (20.36%; green color) and iron (4.49%; pink color) are visible. At a quantity below 1.0%, the following elements are visible: aluminum, sulfur, potassium, magnesium, and carbon. In the event of concrete series S1-1 modified with the waste thermoplastic elastomer in the quantity of 2.5%, the microscopic images present a structure very similar to the reference series concrete (Figure 5). Equally located aggregate particles can be seen in the concrete. EDS analysis of the surface of the S1-1 series concrete shows the presence of calcium (23.48%; blue color), silicon (14.70%; green color), and carbon (4.71%; red color). In addition, the following elements are also present: iron and titanium in quantities of 7.07% and 6.54%, accordingly, as well as aluminum, magnesium, sulfur, potassium, sodium, and zinc in quantities below 1.0%. Similarly, in the event of the cement series S1-4 modified with the waste thermoplastic elastomer in the quantity of 10.0%, a structure very similar to the reference series concrete was observed. EDS analysis shows the presence of calcium (18.98%; blue color), silicon (16.35%; green color), and carbon (9.20%; red color). In addition, the following elements are also present: aluminum and iron in quantities of 1.19% and 1.0%, accordingly, as well as sulfur, magnesium, potassium, and zinc in quantities below 1.0%. The carbon quantity increased by 9.0% in series S1-4, which was caused by the increased quantity of waste in the concrete mixture. The structure of the concrete modified by elastomer waste did not differ significantly from the structure of control concrete. The bending and splitting strength of the concrete containing 2.5% and 5% of waste (amount of carbon < 5%) increased compared to control concrete. On the other hand, a greater amount of waste in the concrete (carbon content of 9.2%) had a negative impact on these strength parameters.

## 4. Conclusions

Tests of the properties and structures of concrete composites containing a waste thermo-plastic elastomer from the production process of car floor mats, as well as analysis of obtained the results, showed the suitability of this waste type for the production of concrete. The early compression strength of concrete (tested after seven days of curing) modified with production waste in the quantity of 2.5% of the cement weight that was used as the substitute for fine gravel aggregate was similar to the compression strength of the reference concrete. On the other hand, in the event of concrete containing waste in the quantities of 5.0%, 7.5%, and 10% of the cement weight, a decrease of a seven-day compression strength was noted at the level between 5.5% to 11.3%. Similarly, the compression strength tested after 28 and 56 days of curing of samples of concrete modified with 2.5% additive of waste was comparable to the strength of the reference samples (increased by 0.3–1.4%). The addition of a higher quantity of waste resulted in a decrease in the values of this parameter by 9.6–22.6% in the event of samples tested after 28 days, and by 11.3–24.4% in the event of samples tested after 56 days of curing. The tests showed that adding a waste thermoplastic elastomer to concrete in the quantities of 2.5% and 5.0% of the cement weight as a substitute for fine gravel aggregate with a particle size distribution 2–8 mm had a positive impact on the increase of the bending strength of concrete. For concrete of these series, an increase in the bending strength by 3.1% and 2.2%, accordingly, as compared to the reference concrete, was noted. Concrete modified with waste in the quantities of 7.5% and 10% showed a decrease in bending strength by 0.8% and 4.2%, accordingly, as compared to the reference concrete. The addition of production waste in the quantities of 2.5% and 5.0% also had a positive impact on the splitting tensile strength of concrete. For these series of concrete, this parameter was higher as compared to the reference concrete by 9.0% and 13.5%, accordingly. In the event of concrete modified with waste in the quantities of 7.5% and 10%, a decrease in the splitting tensile strength of the concrete by 5.3% and 12.3%, accordingly, as compared to the reference concrete, was noted.

All concretes modified with waste obtained lower densities than the reference concrete, which was between 2000 and 2600 kg/m^3^. This allowed for classifying them into the standard concrete category. Both the reference concrete and the concrete modified with waste thermoplastic elastomer obtained absorbability at the ca. 5.0% level. All concretes modified with the waste thermoplastic elastomer met the standard requirements. After 150 cycles of freezing and thawing, the average decrease in compression strength did not exceed 20%, and the average weight loss did not exceed 5%. Therefore, it may be concluded that it is beneficial to add a waste thermoplastic elastomer from the production process of car floor mats in the quantity of 2.5% of the cement weight to concrete, which allows for the saving of natural resources and decreases the costs of concrete manufacture by 20 kg/m^3^, which is about 5.0%.

## Figures and Tables

**Table 1:** Percentage content of the elements in the production waste.

Chemical Composition [% (m/m)]								
Ca	Si	Al	Zn	Mg	S	Ti	Fe	K
2.20	0.49	0.28	0.51	0.10	0.28	0.02	0.02	0.03

**Table 2:** The composition of the reference concrete and concrete modified with waste as the substitute for gravel aggregate with a particle size distribution of 2–8 mm.

Composition	Units	Series				
		SK	S1-1	S1-2	S1-3	S1-4
Cement	kg/m^3^	327.50	327.50	327.50	327.50	372.5
Water	dm^3^/m^3^	167.50	167.50	167.50	167.50	167.5
Sand	kg/m^3^	463.70	463.70	463.70	463.70	463.7
Gravel 8–16	kg/m^3^	776.20	776.20	776.20	776.20	776.2
Gravel 2–8	kg/m^3^	635.10	593.90	552.80	511.70	470.5
Plasticizing admixture	dm^3^/m^3^	1.30	1.30	1.30	1.30	1.30
Air entraining admixture	dm^3^/m^3^	0.745	0.745	0.745	0.745	0.745
Production waste	kg/m^3^	−	9.31	18.63	27.94	37.25

**Table 3:** Consistency class and air content in concrete mixtures subject to tests.

Series	Consistence mm/Class	Air Content [%]
SK	70/S2	3.50
S1-1	55/S2	3.95
S1-2	55/S2	3.90
S1-3	50/S2	3.65
S1-4	75/S2	4.10

**Table 4:** Compression strength of the tested concrete and the resistance class.

Series	Compression Strength [MPa]			Resistance Class
	After 7 Days	After 28 Days	After 56 Days	
SK	46.6	57.0	61.9	C40/50
S1-1	46.5	57.8	62.1	C40/50
S1-2	44.0	51.5	54.9	C35/45
S1-3	42.9	50.1	53.0	C35/45
S1-4	36.1	44.1	46.8	C30/37

**Table 5:** Concrete bending tensile strength and splitting tensile strength.

Series	Bending Strength [MPa]	Splitting Tensile Strength [MPa]
SK	3.59	3.78
S1-1	3.70	4.29
S1-2	3.67	4.12
S1-3	3.56	3.58
S1-4	3.44	3.32

**Table 6:** Parameters tested for each series of concrete.

Series	Water Absorbability [%]	Density [kg/m^3^]	Water Penetration [mm]	Abrasion Strength [cm^2^/50 cm^2^]
SK	5.4	2271	65	7.4
S1-1	5.2	2258	60	6.9
S1-2	5.6	2218	63	6.7
S1-3	5.6	2214	65	7.0
S1-4	5.3	2172	67	7.2

## Data Availability

The data presented in this study are available on request from the corresponding author.
